# 2447. Clinical Features and Outcome of Persistent Candidemia Caused by *Candida albicans* versus Non-*albicans Candida* Species: A Focus on Antifungal Resistance and Follow-up Blood Cultures

**DOI:** 10.1093/ofid/ofad500.2065

**Published:** 2023-11-27

**Authors:** Shiori Kitaya, Hajime Kanamori, yukio Katori, Koichi Tokuda

**Affiliations:** Tohoku University Graduate School of Medicine, Sendai, Miyagi, Japan; Tohoku University Graduate School of Medicine, Sendai, Miyagi, Japan; Tohoku University Graduate School of Medicine, Sendai, Miyagi, Japan; Tohoku University Graduate School of Medicine, Sendai, Miyagi, Japan

## Abstract

**Background:**

Candidemia is a common hospital-acquired (HA) infection linked to extended hospitalization and high mortality rates. Risk factors for persistent candidemia (PC) include central venous catheter, empirical treatment, and metastatic infection foci. Antifungal resistance is a significant global infection control issue, especially in non-*albicans Candida* species. We investigated clinical outcomes and mortality rates associated with HA-PC, focusing on *Candida* species, antifungal resistance, and PC-clearance.

**Methods:**

Data analysis was conducted using electronic medical records from Tohoku University Hospital from 2012 to 2021 (*n* = 60). HA-PC cases were categorized based on *Candida* species, azole or echinocandin resistance, and PC-clearance status, and analyzed respective characteristics. The primary outcome variables were 30-day, 30–90-day, and 90-day mortality after the initial blood culture.

**Results:**

The HA-persistent non-*albicans* candidemia group had a higher ratio of immunosuppression and catheter-related bloodstream infection (CRBSI) than the HA-persistent *Candida albicans* bacteremia group (Odds ratio [OR] = 4.6, *p* = 0.049). CRBSI was more prevalent in the HA-PC resistant strain group than in the susceptible strain group (OR = 4.2, *p* = 0.008), while HA-PC resistant strain group had a lower incidence of intraocular candidiasis (*p* = 0.020). Mortality rates were slightly higher in the HA-persistent non-*albicans* candidemia group compared to the HA-persistent *Candida albicans* bacteremia group. Similarly, the HA-PC resistant strain group tended to have higher mortality rates compared to the susceptible strain group. In the HA-PC non-clearance group, there was a trend towards higher 30-day, and 90-day mortality rates, and the former group had a statistically significant difference (OR = 19, *p* = 0.028).

**Figure:**
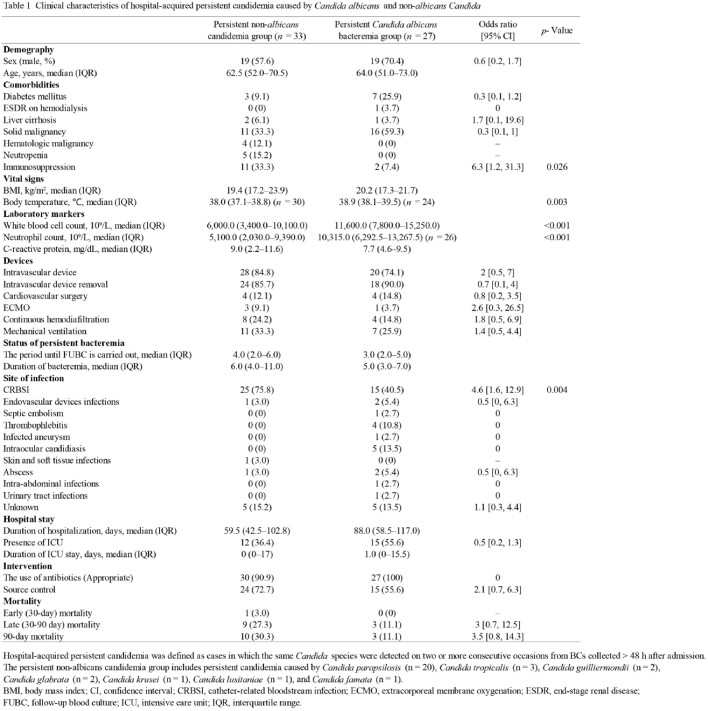

**Figure:**
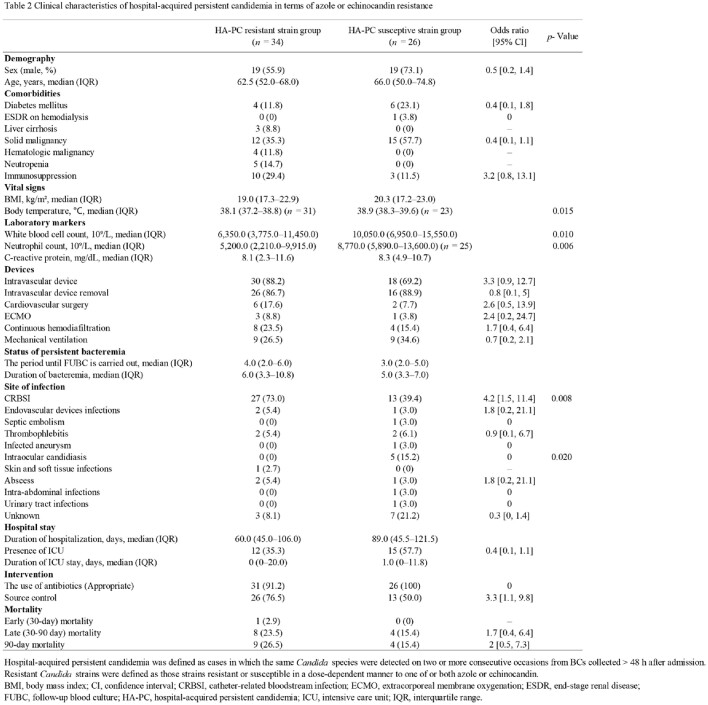

**Conclusion:**

Patients with HA-PC caused by non-*albicans Candida* or azole or echinocandin resistant strains have higher mortality rates and require careful therapeutic management. Confirming the clearance of PC by performing follow-up blood cultures can improve the survival rate for both susceptive and resistant strain groups.

**Disclosures:**

**Shiori Kitaya, MD**, AMANO Co., Ltd.: Grant/Research Support **Hajime Kanamori, MD, PhD, MPH**, Amano Co., Ltd.: Grant/Research Support **Koichi Tokuda, MD, PhD, MPH**, AMANO Co., Ltd.: Grant/Research Support

